# A CMOS Compatible Pyroelectric Mid-Infrared Detector Based on Aluminium Nitride

**DOI:** 10.3390/s19112513

**Published:** 2019-05-31

**Authors:** Christian Ranacher, Cristina Consani, Andreas Tortschanoff, Lukas Rauter, Dominik Holzmann, Clement Fleury, Gerald Stocker, Andrea Fant, Herbert Schaunig, Peter Irsigler, Thomas Grille, Bernhard Jakoby

**Affiliations:** 1 Carinthian Tech Research AG, 9524 Villach, Austria; cristina.consani@ctr.at (C.C.); andreas.tortschanoff@ctr.at (A.T.); lukas.rauter@ctr.at (L.R.); dominik.holzmann@ctr.at (D.H.); clement.fleury@ctr.at (C.F.); 2 Infineon Technologies Austria AG, 9500 Villach, Austria; gerald.stocker@infineon.com (G.S.); andrea.fant@infineon.com (A.F.); herbert.schaunig@infineon.com (H.S.); peter.irsigler@infineon.com (P.I.); thomas.grille@infineon.com (T.G.); 3 Institute for Microelectronics and Microsensors, Johannes Kepler University Linz, 4040 Linz, Austria; bernhard.jakoby@jku.at

**Keywords:** pyroelectric detector, silicon photonics, mid-infrared detector

## Abstract

The detection of infrared radiation is of great interest for a wide range of applications, such as absorption sensing in the infrared spectral range. In this work, we present a CMOS compatible pyroelectric detector which was devised as a mid-infrared detector, comprising aluminium nitride (AlN) as the pyroelectric material and fabricated using semiconductor mass fabrication processes. To ensure thermal decoupling of the detector, the detectors are realized on a Si_3_N_4_/SiO_2_ membrane. The detectors have been tested at a wavelength close to the CO_2_ absorption region in the mid-infrared. Devices with various detector and membrane sizes were fabricated and the influence of these dimensions on the performance was investigated. The noise equivalent power of the first demonstrator devices connected to a readout circuit was measured to be as low as $5.3\times 10^{-9}\;W/\sqrt{\mathrm{Hz}}$.

## 1. Introduction

The detection of infrared radiation is of great interest for a wide range of applications, such as optical gas sensing in the infrared spectral range. In the atmosphere, there are two windows in the infrared spectral region where the absorption due to water vapor and the scattering due to dust are minimal, which are in the range of 3–5μm and 8–14μm [1]. These two transmission windows are of interest for a variety of atmospheric, security and industrial applications with the aim to detect trace concentrations of environmental and toxic gases with sensitivities down to the parts-per-billion range [2].

Furthermore, the peak emission of the black-body curve for objects at 300K is around 10μm. This makes the window from 8–14μm particularly interesting for detection of human beings and other warm-blooded animals [1].

There are mainly two types of infrared detectors, quantum (or photon) detectors and thermal detectors (see e.g., [1,3,4]). In photon detectors, radiation is absorbed due to interaction with electrons. The electrical output signal is the result of a change of the electronic energy distribution. The response per unit incident radiation of photon detectors is wavelength dependent. The advantages of photon detectors is the fast response and the excellent signal to noise ratio. The drawback is that these detectors usually require cooling, which makes them bulky, heavy, expensive and inconvenient to use for certain applications. Thermal detectors are based on the principle that incident radiation, which is absorbed, leads to a change of the temperature. The change in temperature leads to a change in some physical property, which is used to generate an electrical signal. The measurement signal depends on the incident radiant power (or on its rate of change) and not on its spectral distribution. However, the absorption of the radiation can be wavelength dependent and provides design constraints. Depending on the type, different effects are measured, such as the change of the electrical resistance in bolometers, the thermoelectric voltage that is created in a thermocouple, or the change of the internal spontaneous polarization in pyroelectric detectors. Thermal detectors usually operate at room temperature. They show a modest sensitivity and slow response. Due to the fact that a temperature change must be induced by the radiation, thermal detectors need to be thermally decoupled from the surroundings. The advantages are that they are cheap and easy to use [3,4]. Thanks to specific electrical properties, pyroelectric detectors have found a wide range of applications such as thermovision and remote temperature measurement [5]. Furthermore, they perform significantly better than photodetectors at wavelengths higher than 6μm when operated at room temperature [6].

In this work, we investigate a complementary metal–oxide–semiconductor (CMOS) compatible pyroelectric mid-infrared detector for miniaturized gas sensors. Pyroelectricity is defined as the temperature dependence of spontaneous polarization in certain anisotropic solids (see e.g., [1]). It can only occur in materials that have a unique polar axis, creating a current that is proportional to the change in the temperature of the crystal, rather than the temperature itself. A pyroelectric detector is sensitive to radiation that leads to a change in temperature [7], meaning that for sensing applications, it is necessary to use a modulated radiation source. Continuous illumination will only generate a signal until the system is thermally stabilized, therefore the detector does not respond to constant illumination. In contrast to thermocouples, pyroelectric detectors are not thermoelectric.

Pyroelectric materials possess permanent electric dipole moments (see e.g., [1,7]). This means that dipole moments exist in absence of external electric fields. The associated dipole moment per unit volume is called the spontaneous polarization $P_{S}$. A uniformly polarized region features no net polarization charges within the region but only bound (polarization) surface charges at the boundary of aforesaid region. The spontaneous polarization thus corresponds to a layer of bound charge on each surface of the material. If such materials are exposed to a change in temperature, the net dipole moment and consequently the spontaneous polarization changes (decreases for dT/dt>0 and increases for dT/dt<0). If conductive electrodes are attached to the surfaces and are electrically connected, the change of bound charges is compensated by a redistribution of free charges *q* from the electrodes, which results in the pyroelectric current $I_{p}$. In one dimension, the pyroelectric coefficient *p* is defined as

(1) $$p={\left[\frac{\partial P_{S}}{\partial T}\right]}_{\sigma ,E},$$

under the constraints of a constant stress σ and a constant electric field *E*, respectively [1]. The pyroelectric short circuit current is,

(2) $$I_{p}=\frac{dq}{dt}=A\frac{dP_{S}}{dt}=Ap\left[\frac{dT}{dt}\right],$$

where *A* is the area of the detector and dT/dt is the rate of change of temperature (see e.g., [1,7]). A pyroelectric material works as charge generator when subject to a homogeneous change in temperature [7]. Due to the thermal detection approach, pyroelectric detectors only work at low frequencies, which is a disadvantage compared to photon detectors.

Within this work, aluminium nitride (AlN) grown on poly-Si was used as pyroelectric material. In [8], a study on the pyroelectric properties grown AlN on (111)Si was reported. It was shown that the pyroelectric coefficient was practically independent of the temperature and the applied electrical bias and was in the range of $p=6-8\;\mu C/(m^{2}K)$. Furthermore, in [9] a micromachined infrared detector based on an AlN thin film was demonstrated.

**Figure 1:**
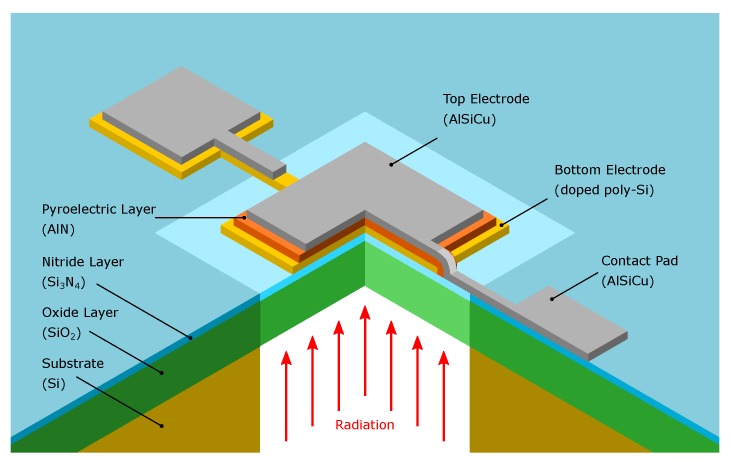
Quarter cut schematic representation of the pyroelectric detector.

In this work, we describe a CMOS compatible mid-infrared detector based on an AlN thin film specifically to be used in a silicon photonics sensor platform that was presented in [10,11]. Our ultimate goal is to fully monolithically integrate a complete optical sensor on a silicon platform. Traditional pyroelectric thermal detectors would not be compatible for this situation, since they usually are designed for free beam illumination from the top, while we expect to couple waveguides to the detector in the same plane. To this aim we developed a novel sensor, using a phosphorous doped-Si layer both as bottom electrode and as infrared absorber. The concept for the fully integrated sensor foresees to have the Si waveguide (not shown in Figure 1), directly connected to the bottom electrode of the detector, meaning that there is a transition of undoped to doped-Si. This approach allows directly coupling the mode that is propagating in the waveguide to the detector. Due to the metal-like properties of highly doped-Si, the mode which is propagating in the waveguide is absorbed in the doped section, which leads to a temperature rise in the pyroelectric AlN layer. To optimize the performance, we investigated various device dimensions, i.e., various combinations of detector and membrane areas. Furthermore, we determined the noise equivalent power of a devised detector system, comprising our best-in-class detector and a custom readout circuit.

## 2. Design

A CMOS compatible pyroelectric detector was designed and realized on a silicon photonics platform. The detector comprises a phosphorous doped poly-Si bottom electrode and an AlSiCu top electrode with an AlN layer sandwiched in between. The poly-Si bottom electrode also acts as infrared radiation absorbing layer. The absorbed radiation induces a temperature change in the poly-Si layer and furthermore also in the AlN layer if the two layers are in sufficient thermal contact. Due to the thermal detection approach, it is crucial to thermally decouple the detector from the substrate, to avoid transfer of the heat to the substrate and maximize the induced temperature change in the AlN layer. Therefore, to achieve the thermal decoupling from the substrate, the devised detector is located on a free-standing Si_3_N_4_/SiO_2_ membrane. To electrically connect the detector, both electrodes feature the necessary contact pads. Figure 1 shows a schematic quarter cut of the devised detector.

The SiO_2_ layer that is located below the Si_3_N_4_ layer is necessary for the evanescent field absorption sensing, using a Si waveguide in order to suppress coupling of an electromagnetic mode into the substrate. In the final application, for further thermal decoupling, it would be beneficial to remove the SiO_2_ layer below the detector area as this is expected to result in a higher response of the detector. Within this work various detector/membrane dimensions were tested. The size of the membrane was varied from $0.14\;{\mathrm{mm}}^{2}$ to $1.44\;{\mathrm{mm}}^{2}$ and the size of the detector (more specifically of the AlN pad) was varied from $0.03\;{\mathrm{mm}}^{2}$ to $1\;{\mathrm{mm}}^{2}$. For configurations with large membrane, it was not foreseeable whether a membrane that is only comprised of the thin Si_3_N_4_ membrane would be mechanically stable and therefore, the SiO_2_ layer was not removed, leading to a Si_3_N_4_/SiO_2_ membrane.

## 3. Experimental

### 3.1. Fabrication of the Detector

The designed detector was fabricated on eight inch wafer, using standard industrial CMOS production processes such as chemical vapor deposition, lithography and dry etching. Below the Si_3_N_4_ layer, a SiO_2_ oxide layer with a thickness of 2μm was deposited at a temperature of $670{\;}^{\circ }C$. The Si_3_N_4_ layer on which the detector is located is deposited on top of the oxide as a Si_3_N_4_ layer with a thickness of 140nm. As described above, the concept intends to absorb a propagating mode in a waveguide in a section which is doped with phosphor. Therefore, amorphous Si was deposited using low pressure chemical vapor deposition, annealed ($700{\;}^{\circ }C$ for 30s to achieve a poly-crystalline Si layer), structured using a dry etching process and doped with phosphor ($\mathrm{dose}=\;3.60\times 10^{15}\;{\mathrm{cm}}^{-2}$). The pyroelectric AlN layer was deposited using a sputtering process with a thickness of 500nm and structured using a dry etching process. The AlSiCu top electrode (1000nm) was deposited using a sputtering process and structured using a wet-chemical etch process. To reduce the thermal mass, the silicon substrate was removed underneath the detector, using a deep reactive ion etching process from the backside.

### 3.2. Characterization of the Detector

**Figure 2:**
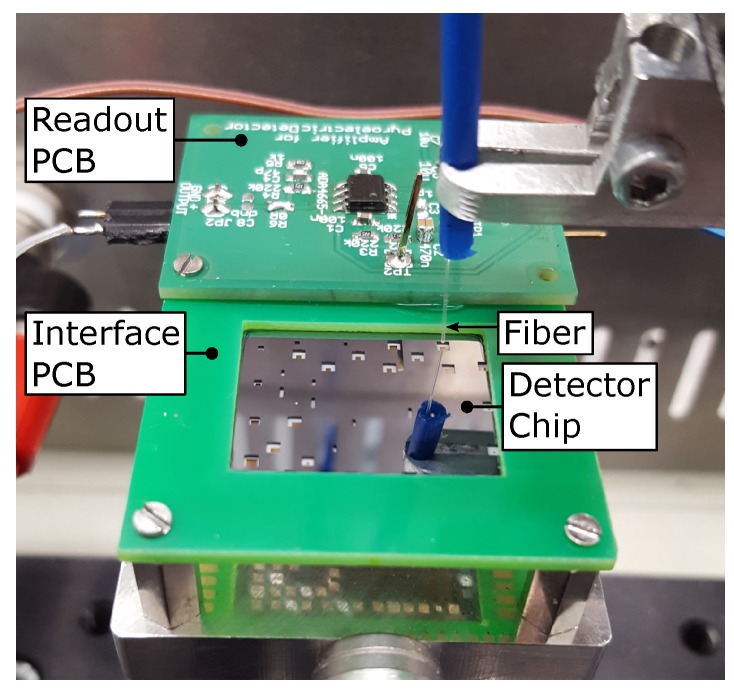
A section of the measurement configuration, depicting the backside of a detector chip which is mounted in the interface PCB, together with the attached readout PCB and the optical fiber. The optical fiber is approached to the backside of a detector.

The measurement configuration is shown in Figure 2. The diced detector chips were glued onto an interface printed circuit board (interface PCB) which was used as interface between the chip and the readout circuit. The contact pads of the detector were wire bonded to the interface PCB, using a Kulicke & Soffa Model 4526 wire bonder and an Au wire. The bonded chip was flipped upside down and optionally, a readout circuit PCB was glued and wired on the backside of the interface PCB (see Figure 2).

The topography of the fabricated detectors was investigated using a Thermo Fisher Helios G4 UC scanning electron/focused ion beam microscope. The performance of the detectors was characterized, using a custom test-bench that comprises an optical setup, including a quantum cascade laser (MIRcat laser system from Daylight Solutions) as well as a grounded metal box in which the detector system is located. The quantum cascade laser (QCL) was operated in continuous wave mode and was modulated using Stanford Research SR540 chopper controller for variable frequencies, whereas a frequency of roughly 6Hz was used. This is the lowest frequency at which the used chopper is sufficiently stable. Furthermore, a single frequency chopper (Ophir RMC1 Chopper) was used, which modulates the laser precisely at a frequency of 18Hz, with a duty cycle of 50%. The choice of 6Hz and 18Hz is arbitrary and not related to the characteristics of the devised detector. The laser radiation was coupled into an optical fiber (Thorlabs MF11 with a core diameter of 100μm) and guided to the backside hole of the detector under test. The illumination of the detector is performed from the bottom side (i.e., the radiation is striking the SiO_2_ layer first) in order to avoid strong reflections at the metal top electrode, which would decrease the amount of radiation that can reach the doped-Si layer and be absorbed. The laser power at the output of the fiber was measured, using a thermopile detector powermeter (Newport Power Meter Model 1919-R with Thermopile detector 919P-003-10) and a pyroelectric detector powermeter (Vega Ophir), respectively. During the measurements, the metal box was closed with a grounded metal cover in order to reduce external noise.

**Figure 3:**
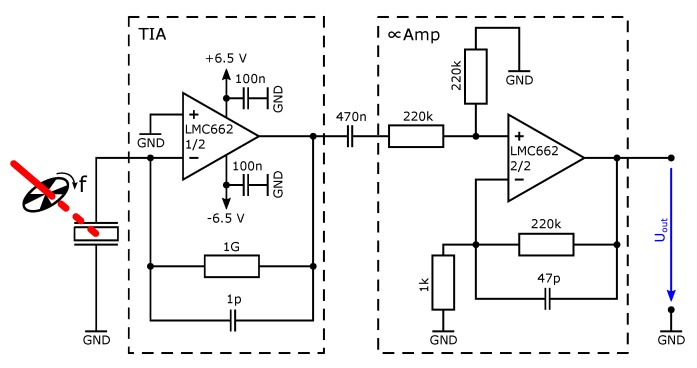
A scheme of the readout circuit for the pyroelectric detector. The modulated radiation that hits the pyroelectric detector leads to a generation of free charges.

For the investigation that aimed to determine the best performing device, a simple readout approach was used, which did not require connecting a readout circuit PCB to the interface PCB. The generated current was directly measured, using the current input (1kΩ) of a lock-in amplifier (Stanford Research SR830m). In contrast, the investigation of the noise equivalent power was conducted using the detector with the additional readout PCB. The readout circuit comprised a transimpedance amplifier and a non-inverting proportional amplifier with filters. A schematic representation of the detector with the readout circuit is shown in Figure 3. The output of this circuit as well as the clock signal of the chopper was recorded using an oscilloscope (Picoscope 5444B).

For the characterization of the noise equivalent power, optical reflection filters from Thorlabs, namely NDIR03B (OD=0.3), NDIR10B (OD=1.0) and NDIR20B (OD=2.0) have been used to modify/attenuate the optical power that reaches the detector. The optical setup was adjusted to a optical power at the output of the optical fiber, which was at the low measurement range of the power meter while the OD=2.0 filter was inserted in the setup, leading to an optical power at the output of the optical fiber of $P_{0}=1.5\;\mu W$. Furthermore, higher incident power was then achieved by using the OD=1.0 and OD=0.3 filters, which led to $P_{0}=16.3\;\mu W$ and $P_{0}=70.2\;\mu W$.

## 4. Results and Discussion

**Figure 4:**
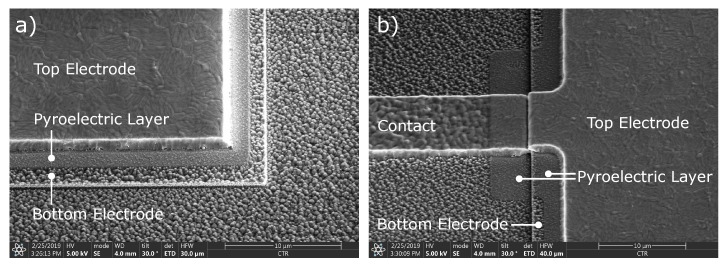
SEM micrograph of a fabricated detector. (**a**) The bottom-right part of the detector, depicting the different layers, the bottom electrode (doped-Si), the pyroelectric layer (AlN) and the top electrode (AlSiCu) of the detector. Due to difficulties of the AlN patterning, the bottom electrode is covered with grainy residues of AlN. (**b**) the most left-middle part of the detector, depicting how the top electrode is contacted.

The topography of the fabricated structures has been investigated using scanning electron microscopy. A micrograph of the bottom-right part and the middle-left part of a detector is shown in Figure 4a,b, respectively. Due to difficulties during the patterning of the AlN layer, a rough grainy structure of the AlN layer is visible in the areas where it should have been removed. Therefore, residues of AlN are also most probably between the doped-Si contact pad and the AlSiCu layer, which will therefore supposedly influence the contact resistance. This would provide a spurious capacitance in series to the capacitance of the detector, which, however, is not necessarily detrimental for the charge transfer in AC.

**Figure 5:**
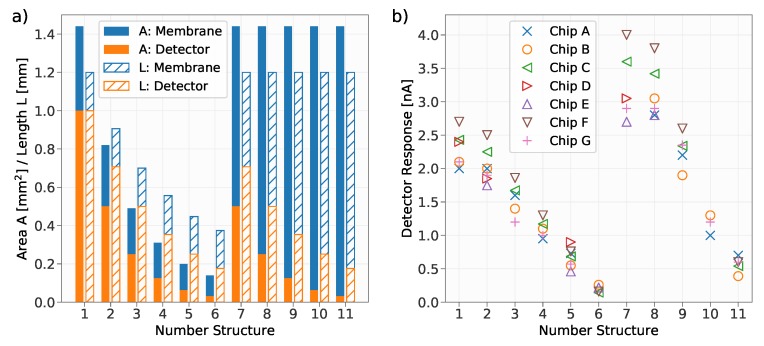
(**a**) The area and the lateral length of the various tested detector designs. Both the detector and the membrane are quadratic. (**b**) The generated current of the investigated detectors when irradiated with a power of 11mW at a wavelength of 4.17μm and modulated at a frequency of 6Hz.

When exposed to a modulated radiation, the pyroelectric detector creates a current according to Equation (2). These experiments were conducted at a wavelength of 4.17μm, which is just outside of the mid-infrared absorption band of CO_2_ in order to avoid an influence of the ambient CO_2_ concentration. The detector and membrane areas of the investigated structures are shown Figure 5a. The output current of the detectors was measured using the lock-in amplifier. Each detector was irradiated with a power of 11mW modulated at a frequency of 6Hz. The measured current of the detectors is shown in Figure 5b.

For this investigation, in total seven detector chips (labelled *A* to *G*) have been characterized. As shown in Figure 5b, there is some data missing, which is due to the fact that not every detector worked due to failures such as broken detector membranes. It can be observed that the detectors #7 and #8 with a detector area of $0.50\;{\mathrm{mm}}^{2}$ and $0.25\;{\mathrm{mm}}^{2}$, respectively and a membrane size of $1.44\;{\mathrm{mm}}^{2}$ showed the largest response. The measurements show that the highest response, among the tested configurations, can be achieved for detectors with a large membrane area and a small detector area. Obviously, the choice of the irradiated area (i.e., choice of the diameter of the optical fiber and its distance to the detector) also has an influence on the heating of the detector and therefore on the measured current. This effect was not further investigated within this work.

**Figure 6:**
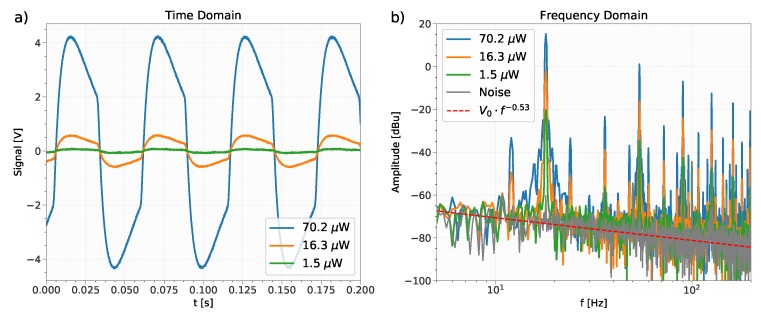
The voltage response of the pyroelectric detector is exposed to modulated infrared radiation $P_{0}$. (**a**) Time domain signals. (**b**) Frequency domain signals together with a recorded noise signal (i.e., no incident radiation), the $f^{-0.53}$ decrease in the amplitude corresponds to a PSD dependence in $f^{-1.05}$.

For further investigations, four different detector chips (labelled *H* to *K*) were used. The experiments described below were conducted using the detector structure #8 and the readout circuit presented in Figure 3. The detector response is measured as a voltage. Representative sections of recorded detector response signals to incident radiation $P_{0}$ (70.2μW, 16.3μW and 1.5μW) are shown in Figure 6a. We note that the optimal modulation frequency (i.e., frequency with highest root mean square (RMS) current output) for the device #8 was also determined using the experimental setup of the previous experiment and was 31±4Hz. Nevertheless, for these measurements, the chopper which modulates the signal at a frequency of precisely f=18Hz with a duty cycle of 50% was used in order to allow for accurate data analysis. Although the modulation frequency of 18Hz was too high to reach a steady state of the detector response within the “on” and “off” periods of the QCL, it can be observed that the detector shows the expected response to the rectangular modulated radiation (compare with, e.g., [5]). The measured voltage changes sign according to the derivative of the temperature (Equation (2)). A fast Fourier transform (FFT) analysis has been conducted and the lower frequency section of the results is shown in Figure 6b in units of dBu, showing the fundamental peak at 18Hz. Here also, a measurement of the noise (no incident radiation) is presented. The time domain signals were recorded for 4.5s with 100kS. The measurements were conducted for two amplifier boards with two chips (using the chips *J* to *G*) each, which are identical in design, leading to four measurement configurations. The measurements were performed using three different input radiation powers for each configuration and an additional noise measurement. Each measurement was repeated three times. From the frequency spectrum, the peak value of the amplitude (in units of V) at f=18Hz was determined for each measurement. The analysis of the power spectral density (PSD) of the noise (the PSD is not plotted in Figure 6b) between 10 and 45Hz shows that the noise behaves as a pink noise with an exponent of −1.05. Beyond 500Hz, the noise is roughly flat except for spikes that can be attributed to the environment. The noise in this range is therefore probably dominated by the instrumentation (readout circuit plus oscilloscope). The responsivity of the sensor at 18Hz was computed as the ratio

(3) $$R_{V,18\mathrm{Hz}}=\frac{V_{S,18\mathrm{Hz}}}{P_{0,18\mathrm{Hz}}}=\frac{\pi \cdot V_{S,18\mathrm{Hz}}}{2\cdot P_{0}},$$

where $V_{S,18\mathrm{Hz}}$ and $P_{0.18\mathrm{Hz}}$ are the amplitudes of the signal and of the incident power at the fundamental frequency, respectively. The factor 2/π is the first Fourier coefficient of the optical power square wave. The incident power at the fundamental peak can be calculated as $P_{0,18\mathrm{Hz}}=2/\pi \cdot P_{0}$.

**Figure 7:**
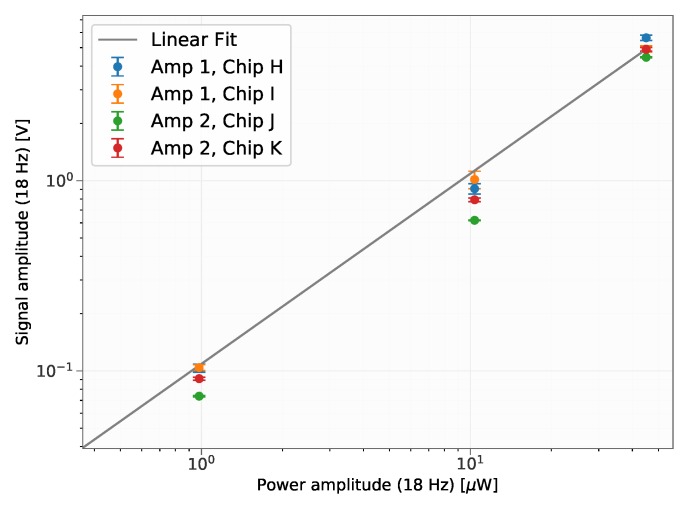
The response of the detector (amplitude at 18Hz, $V_{S,18\mathrm{Hz}}$) as a function of the incident power at 18Hz ($P_{0,18\mathrm{Hz}}$) that was irradiated onto the detector. The fit was conducted using the entire data set shown in the plot. It was also repeated for the specific data associated with each individual configuration, which led to virtually the same result for the response $R_{V}$ when taking the mean value of the individual results.

Figure 7 shows the amplitude of the signal $V_{S,18\mathrm{Hz}}$ versus the incident power for a modulation frequency of 18Hz ($P_{0.18\mathrm{Hz}}$). The data was linearly fitted to get the responsivity $R_{V}=1.09\times 10^{5}\;V/W$. The noise equivalent power (NEP) can be computed from the noise level in a bandwidth of B=5Hz around 18Hz, $V_{N}$ and the responsivity $R_{V}$ as (see e.g., [1])

(4) $$N\;E\;P=\frac{V_{N}}{\sqrt{B}\cdot R_{V}}=\frac{\sqrt{\sum_{B}\left(PSD\right)}}{\sqrt{B}\cdot R_{V}}.$$

The NEP refers to optical power incident on the pyroelectric detector system and is therefore known as optical NEP [12]. Furthermore, the detectivity $D^{*}$ is defined as

(5) $$D^{*}=\frac{\sqrt{A}}{N\;E\;P},$$

where *A* is the area of the detector, which was $0.25\;{\mathrm{mm}}^{2}$ for the investigated detector #8. The device cut-off frequency was estimated from a simulation of the readout circuit to be $f_{C}=200\;\mathrm{Hz}$, using the Qucs software. Using the determined parameters, the noise equivalent power of the detector system at 18Hz was calculated to be $N\;E\;P=5.3\times 10^{-9}\;W/\sqrt{\mathrm{Hz}}$ and the detectivity as $D^{*}=9.4\times 10^{6}\;\mathrm{cm}\sqrt{\mathrm{Hz}}/W$. These parameters correspond to the performance of the investigated demonstrator detector system, meaning the detector together with the readout circuit and furthermore assuming that the whole radiation leaving the optical fiber hits the detector. For comparison, a commercially available detector (Infratec LME-351) which is based on the pyroelectric material lithium-tantalate, reaches a detectivity of $D^{*}=1.8\times 10^{8}\;\mathrm{cm}\sqrt{\mathrm{Hz}}/W$ ($A=4\;{\mathrm{mm}}^{2}$) according to the datasheet [13]. In [14] a review of CMOS compatible thermopile infrared detectors has been presented, reporting detectvities up to the tens of $10^{8}\;\mathrm{cm}\sqrt{\mathrm{Hz}}/W$ range. In [15] a CMOS compatible n-well microbolometer array with a detectivity of $D^{*}=2.6\times 10^{8}\;\mathrm{cm}\sqrt{\mathrm{Hz}}/W$ ($A=4\;{\mathrm{mm}}^{2}$) has been presented. Although the performance of the devised demonstrator devices does not yet reach the performance of previously reported thermopiles and bolometers, nor does it reach the performance of commercially available pyroelectric detectors, the results are very promising. In particular, while the detector was characterized using backside illumination (on the doped poly-Si electrode), we shall keep in mind that in the final scheme the detector will be illuminated from the side using silicon waveguides. If using the bottom electrode as the light absorber is attractive due to the easy integrability with waveguides, the concept comes with limitations in the choice of the absorber material, due to compatibility with the subsequent processes. In contrast to other pyroelectric detectors, absorption does not occur in a very thin (black) metal layer deposited on the top or in complex plasmonic and/or metamaterial absorbers (see e.g., [7,16,17,18]). As a consequence, the responsivity of the detector is not as high as that of commercial devices. Nevertheless, the concept is particularly promising due to the high integrability with photonics structures, and has the potential to be further extended by using metamaterial designs in order to enhance light absorption at the desired wavelengths. Finally, there is still a margin to improve the performance of the readout electronics.

## 5. Conclusions

A CMOS compatible pyroelectric detector based on an AlN thin film was demonstrated. Various dimensions concerning the detector area and the membrane area on which the detector was located were investigated. Among the tested ones, the highest response was achieved with a detector area of $0.25\;{\mathrm{mm}}^{2}$ and $0.50\;{\mathrm{mm}}^{2}$, respectively and a membrane size of $1.44\;{\mathrm{mm}}^{2}$. The noise equivalent power of the detector with an area of $0.25\;{\mathrm{mm}}^{2}$ was investigated and was as low as $N\;E\;P=5.3\times 10^{-9}\;W/\sqrt{\mathrm{Hz}}$ for the investigated demonstrator devices and a measurement bandwidth of 1Hz, which corresponds to a detectivity of $D^{*}=9.4\times 10^{6}\;\mathrm{cm}\sqrt{\mathrm{Hz}}/W$.
